# Network-Based Pharmacological Study on the Mechanism of Guishao-Liujun Decoction in the Treatment of Gastric Cancer

**DOI:** 10.3389/fphar.2022.937439

**Published:** 2022-07-05

**Authors:** Xiaoqing Qian, Lingle Zhang, Feng Xie, Yingsheng Cheng, Daxiang Cui

**Affiliations:** ^1^ School of Biomedical Engineering, Shanghai Jiao Tong University, Shanghai, China; ^2^ Institute of Nano Biomedicine and Engineering, Shanghai Engineering Research Centre for Intelligent Diagnosis and Treatment Instrument, Department of Instrument Science and Engineering, School of Electronic Information and Electrical Engineering, School of Sensing Science and Engineering, Shanghai Jiao Tong University, Shanghai, China; ^3^ College of Fisheries and Life Science, Shanghai Ocean University, Shanghai, China; ^4^ Department of Thoracic Surgery, Renji Hospital, School of Medicine, Shanghai Jiao Tong University, Shanghai, China; ^5^ Department of Radiology, Shanghai Jiao Tong University Affiliated Sixth People’s Hospital, Shanghai, China

**Keywords:** network pharmacology, macromolecular docking, mechanism of action, GC, Guishao-Liujun decoction

## Abstract

**Objective:** The aim of the study was to use a network pharmacological method to examine the mechanism of Guishao-Liujun decoction against gastric cancer (GC).

**Methods:** The traditional Chinese medicine systems pharmacology database and analysis platform (TCMSP) and the Traditional Chinese Medicine Integrated Database (TCMID) were used to obtain the chemical composition and targets of all the drugs of Guishao-Liujun decoction, and the targets of GC were screened using GeneCards and Online Mendelian Inheritance in Man (OMIM) databases. The obtained targets were imported into Cytoscape 3.7.2 software by using the R language to take the intersection for a Venn analysis to construct active ingredient target networks, and they were imported into the STRING database to construct protein–protein interaction (PPI) networks, with the BisoGenet plugin in Cytoscape 3.7.2 being used for analyzing network topology. On the potential target of Guishao-Liujun decoction for GC, gene ontology (GO) enrichment analysis and Kyoto Encyclopedia of Genes and Genomes (KEGG) enrichment analysis were performed using the R-language bioconductor platform, and the outcomes were imported into Cytoscape 3.7.2 software to obtain the KEGG network map. The core targets were docked with the active components by the macromolecular docking software application AutoDock Vina.

**Results:** A total of 243 chemical components and 1,448 disease targets including 127 intersecting targets were discovered. AKT1, TP53, and GO functional analysis were mainly associated with ubiquitination and oxidase reduction activity. In GC treatment, the KEGG analysis revealed that Guishao-Liujun decoction mainly acted through the tumor necrosis factor (TNF), interleukin 17 (IL-17), and cancer-related signaling pathways, with the best binding performance with TP53, as indicated by the outcomes of macromolecular docking.

**Conclusion:** In the treatment of GC, Guishao-Liujun decoction works with a variety of components and targets, establishing the groundwork for further research into its mechanism of action.

## Introduction

Over the past 40 years, China has experienced rapid demographic and epidemiological changes. Rapid industrialization, urbanization, aging, and lifestyle changes have shifted the burden of the disease spectrum from infectious to non-infectious diseases. With a large population, China plays an important role in the global cancer burden (He et al., 2021). Gastric cancer is common all over the world. One in 78 women and one in 33 men have gastric cancer for their whole life (GBD 2017 Causes of Death Collaborators, 2018). According to GBD 2019, the DALYs in China accounted for 44.21% of the total number (GBD 2019 Diseases and Injuries Collaborators, 2020). The causes of GC are relatively complicated and are closely linked to dietary conditions and geographical environments along with infection by *Helicobacter pylori* (*H. pylori*) and genetic factors. GC is relatively insidious, and most of the affected people are diagnosed in the middle and late stages. Presently, the key treatment of GC is still surgery, but patients face many problems post-surgery including immune dysfunction and the slow recovery of digestive tract function, which has a serious impact on the life quality and clinical efficacy of patients (Disbrow et al., 1993). Research has revealed that Guishao-Liujun decoction improves gastrointestinal function in a better manner and enhances the immune function of GC patients (Zhou et al., 2022).

The ingredients of Guishao-Liujun decoction include *Poria cocos* (Schw*.*)Wolf, *Codonopsis* Radix, *Herba Hedyotis diffusa*, Largehead Atractylodes Rhizome, *Salvia chinensis* Benth., Radix Aucklandiae, *Angelica sinensis*, *Pinellia ternata*, *Sparganium stoloniferum* (Graebn*.*) Buch*. -Ham. ex Juz.*, *Paeonia lactiflora* Pall*.*, *Curcuma phaeocaulis* Valeton, *Citrus reticulata* Blanco, *Amomum villosum* Lour*.*, *Roasted Licorice,* and *Radix Glycyrrhizae Preparata*, which nourish the qi and blood, treat the deficiencies of the spleen and stomach, and improve swelling and abdominal fullness. At present, most reports have studied Guishao-Liujun decoction’s mechanism of action in the treatment of GC through clinical observation, and pharmacological studies are lacking on the use of Guishao-Liujun decoction for the treatment of GC as a result of the diversity of GC genes and the complexity of herbal components (Zhang et al., 2019a). Furthermore, this research has used a network of pharmacological methods to examine the mechanism of action of Guishao-Liujun decoction in the treatment of GC.

## Materials and Methods

### Screening of Target Components of Guishao-Liujun Decoction

The TCMSP and TCMID pharmacology platform of the traditional Chinese medicine system was used to search for the active chemical components of the Chinese herbs (*Poria cocos* (Schw*.*)Wolf, *Codonopsis* Radix, *Herba Hedyotis diffusa*, Largehead Atractylodes Rhizome, *Salvia chinensis* Benth*.*, Radix Aucklandiae, *Angelica sinensis*, *Pinellia ternata*, *Sparganium stoloniferum* (Graebn*.*) Buch*. -Ham. ex Juz.*, *Paeonia lactiflora* Pall*.*, *Curcuma phaeocaulis* Valeton., *Citrus reticulata* Blanco., *Amomum villosum* Lour*.*, *Roasted Licorice* and Radix *Glycyrrhizae Preparata*) in Guishao-Liujun decoction. For screening purposes, the TCMSP was set with an oral bioavailability (OB) ≥ 30% and drug-likeness (DL) ≥ 0.18 (Zhang et al., 2019b), the TCMID was screened in SwissADME, and screening conditions were set as follows: gastrointestinal (GI) absorption was enhanced in pharmacokinetics and more than two yeses in drug-likeness to filter out ineffective components and collect the active ingredients in Guishao-Liujun decoction. The SwissTargetPrediction database was used to explore the possible protein targets with the screening condition probability set at ≥ 0.1. The UniProt database was used to convert screened protein targets into standardized gene names.

### Screening of GC-Related Targets

To find target genes linked to GC, the term “gastric cancer” was searched on the OMIM and GeneCards databases. Because of a large number of targets in the GeneCards database, the targets were filtered according to their score value. The higher the score value, the stronger will be the link between the target and the disease, and the target with a score higher than the median is usually set as a potential disease target, leaving the genes with a score higher than five in the GeneCards to be combined and de-weighted with the OMIM database, which is the target associated with GC.

### Acquisition of Effective Targets and Drawing of the Venn Diagram

A Venn diagram was used to obtain an intersection of the target points of Guishao-Liujun decoction and GC’s target points, and the intersection of the two targets was indicated to be the effective target of Guishao-Liujun decoction for GC treatment.

### Active Ingredient-Active Target Network Construction Analysis

The active components and effective target genes were imported into Cytoscape 3.7.2 (Zhang et al., 2019a) software for developing a network and to carry out a visualization analysis to get a drug-active ingredient-target network diagram, and the importance of the active component and its target of action were assessed using analyzing network topological parameters such as degree values.

### Construction of Protein Networks

We imported the effective targets of Guishao-Liujun decoction and GC into the STRING database; then we constructed a protein interaction network and selected data with confidence ≥ 0.900. The results were imported into Cytoscape 3.7.2 software (Martin et al., 2010) in tab-separated value (TSV) format so that we can analyze and visualize them. The PPI data were imported with R software to obtain the number of connection points of core genes, and the graph was obtained for the histogram’s top 30 core genes.

### Enrichment Analysis of Target Functions and Pathways

We collected gene IDs (entrezID) of potential targets using the R software (https://www.r-project.org/) and its backend database org. Hs.eg.db. The GO function enrichment analysis of the possible targets was conducted with the Disease Ontology Semantic and Enrichment analysis (DOSE), cluster profile, and path view package (Bioconductor), comprising the following three aspects: biological process (BP), cellular component (CC), and molecular function (MF), set to *p*-value cutoff = 0.05 and q-value cutoff = 0.05.

The GO enrichment analysis was categorized into three main groups, namely, biological process (BP), molecular function (MF), and cellular component (CC). Each group was ranked by significance, and bar and bubble charts were used to illustrate the top 10 enrichment entries.

### Macromolecular Docking of the Main Active Ingredients and Targets of Guishao-Liujun Decoction

The targets of Guishao-Liujun decoction working on GC were found in the Protein Data Bank (PDB) database, and we saved the data in the PDB format. The mol2 format was used to save ligands with the top two compounds in terms of degree value after topological analysis. We carried out molecular docking between the potential targets of Guishao-Liujun decoction in GC and the main compounds in Guishao-Liujun decoction using AutoDockTools-1.5.6.

## Results

### Acquisition of Active Ingredients and Related Targets of Guishao-Liujun Decoction

With OB 30% and DL 0.18, GI absorption as high in pharmacokinetics, and more than two yeses in drug-likeness as screening conditions, a total of 181 active ingredients in Guishao-Liujun decoction were obtained in the TCMSP and TCMID, respectively, and some of the active ingredients are listed in Supplementary Table S1. The TCMSP and TCMID gave us with 243 TCM targets.

### Acquisition of GC-Related Targets

GeneCards and OMIM provided the affected genes, and targets with scores higher than the median were picked as prospective disease targets empirically. A total of 1,448 GC-related targets were obtained after combining the relevant targets retrieved from the OMIM database, merging, and deleting duplicate values.

### Venn Drawing

**FIGURE 1:**
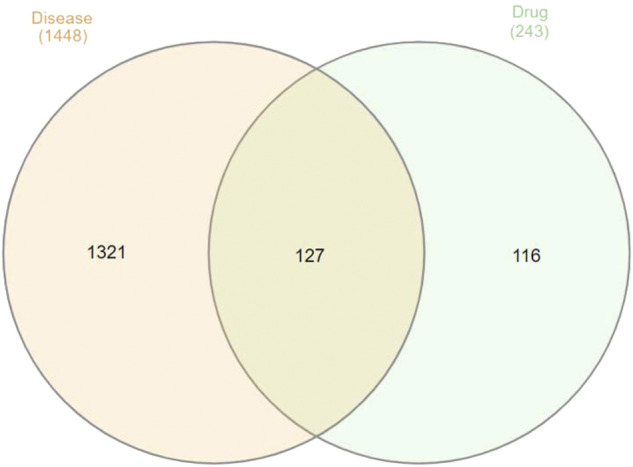
Venn diagram of the intersectional targets of Guishao-Liujun decoction and GC.

A total of 127 intersection targets of the aforementioned were obtained using the Venn diagram tool to take the intersection of Guishao-Liujun decoction and GC targets, and the results are illustrated in Figure 1.

### Construction of the Active Ingredient–Effective Target Network Diagram of Guishao-Liujun Decoction

**FIGURE 2:**
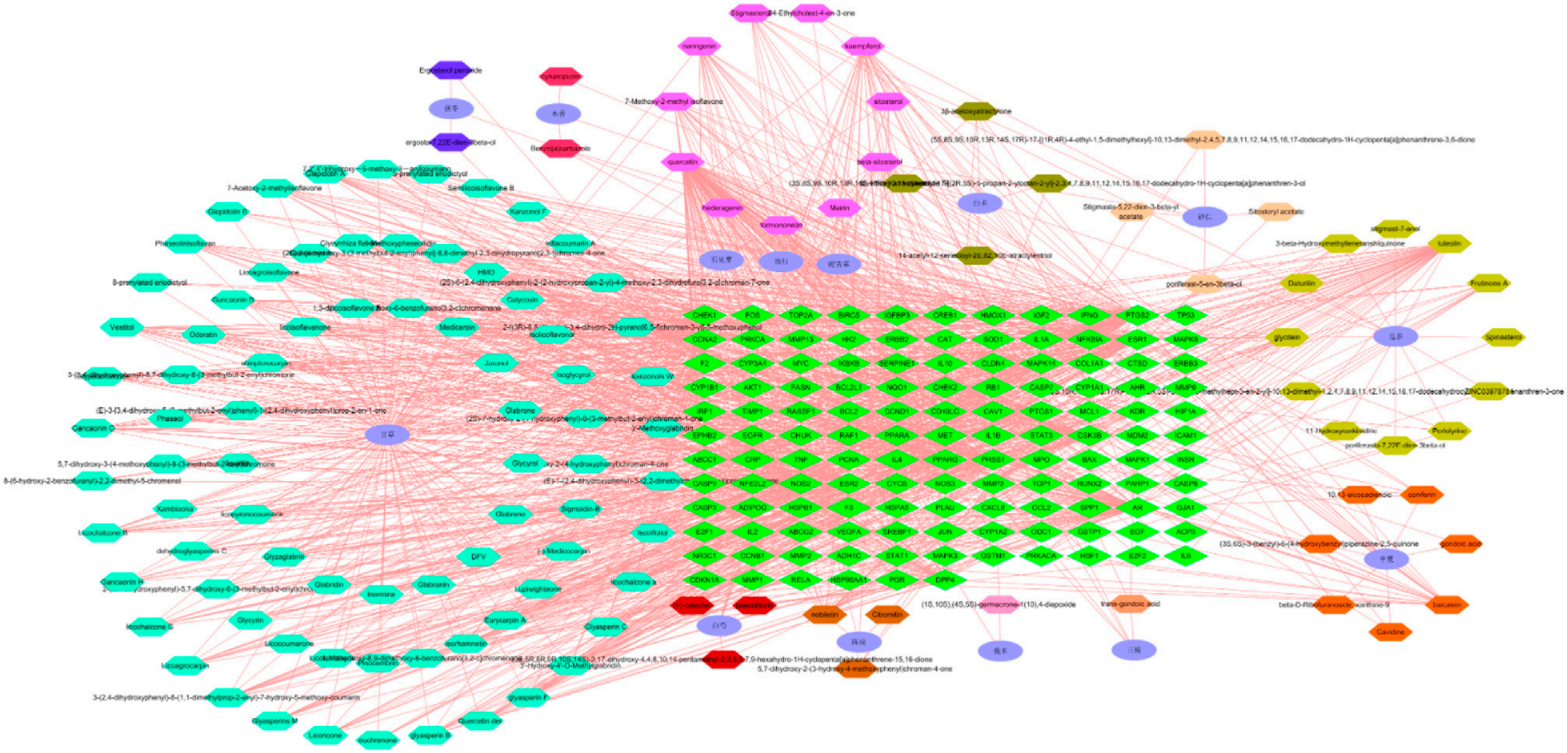
Active ingredient–active target network of Guishao-Liujun decoction.

The network of active ingredients and effective targets of Guishao-Liujun decoction was built using Cytoscape 3.7.2, which is shown in Figure 2. Its topological parameters for GC were measured by the software, which were used to assess the significance of the active components and targets of action. The findings demonstrated that the active ingredients such as kaempferol, quercetin, luteolin, and decursinol angelate had the potential to act on more than one target, and they might be the active ingredients of Guixia Shao Liujun Tang that play a major role in GC treatment.

### Construction of Protein Networks

**FIGURE 3:**
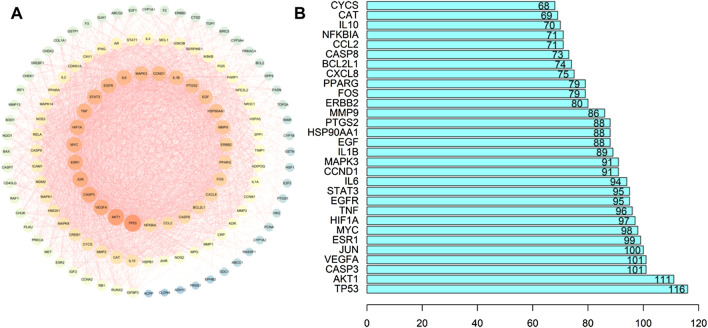
Intersection target PPI network diagram. **(A)** GO enrichment analysis; **(B)** core genetic map.

Using the Venn tool in TBtools (Figure 1), the intersection of the targets of Guishao-Liujun decoction and the targets of GC were obtained and the intersection targets were uploaded to the STRING database with a confidence level of ≥ 0.9 to obtain the PPI network diagram of the targets. Then the existing data were imported into Cytoscape 3.7.2 to plot protein network relationships; the larger the node, the larger will be the degree value. The location in the network was judged on the basis of the degree value. As shown in Figure 3, the targets in the center of the network are TP53, AKT1, Caspase-3 (CASP3), vascular endothelial growth factor A (VEGFA), etc., which are supposed to be the significant targets for GC treatment using Guishao-Liujun decoction.

### Results of Enrichment Analysis of Target Functions and Pathways

**FIGURE 4:**
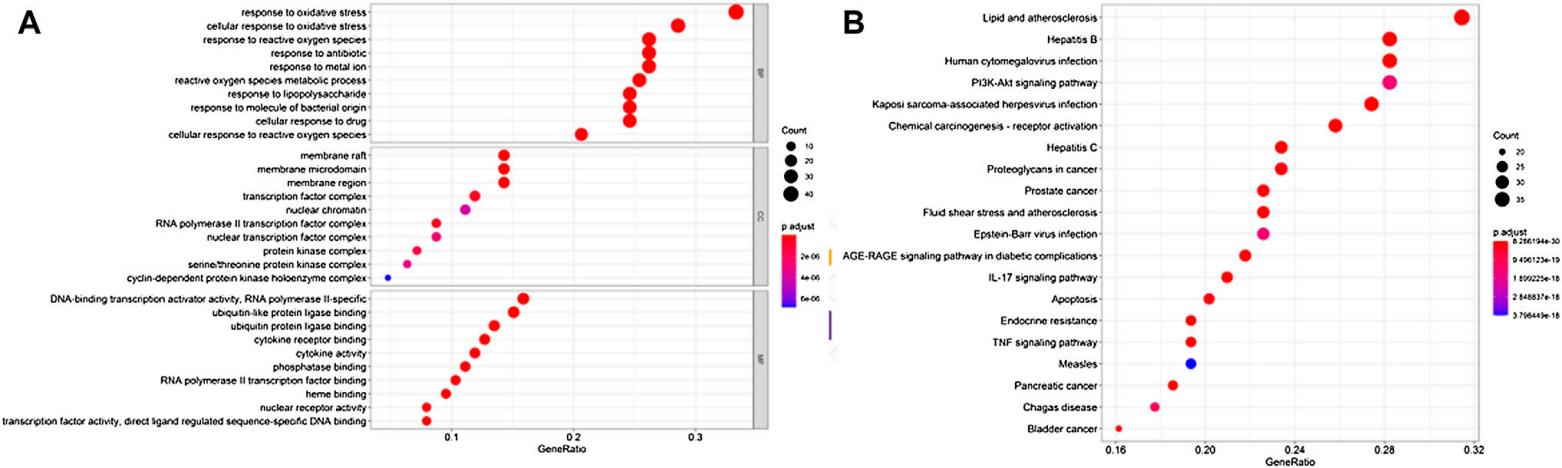
Enrichment analysis of Guishao-Liujun decoction for GC. **(A)** GO enrichment analysis; **(B)** KEGG enrichment analysis.

R was used for the GO annotation analysis of the effective targets. We picked the top 20 BP, CC, and MF results, and found that these BP targets were mostly involved in oxidative stress and cellular oxidative stress, metal ion response, antibiotic response, etc. MF was primarily involved in the binding of ubiquitinated protein ligase, cytokine response, and phosphorylase. CC was mostly involved in the membrane micro-domain, membrane rafts, transcription factor complexes, chromatin, etc. The results are shown in Figure 4A.

KEGG enriched 179 signaling pathways such as the TNF signaling pathway, P13K-Akt, IL-17 signaling pathway, mitogen-activated protein kinase 8 (MAPK8), etc. The top 20 pathways were visualized, and the results are shown in Figure 4B.

### Macromolecular Docking Results of the Active Ingredients of Guishao-Liujun Decoction

**FIGURE 5:**
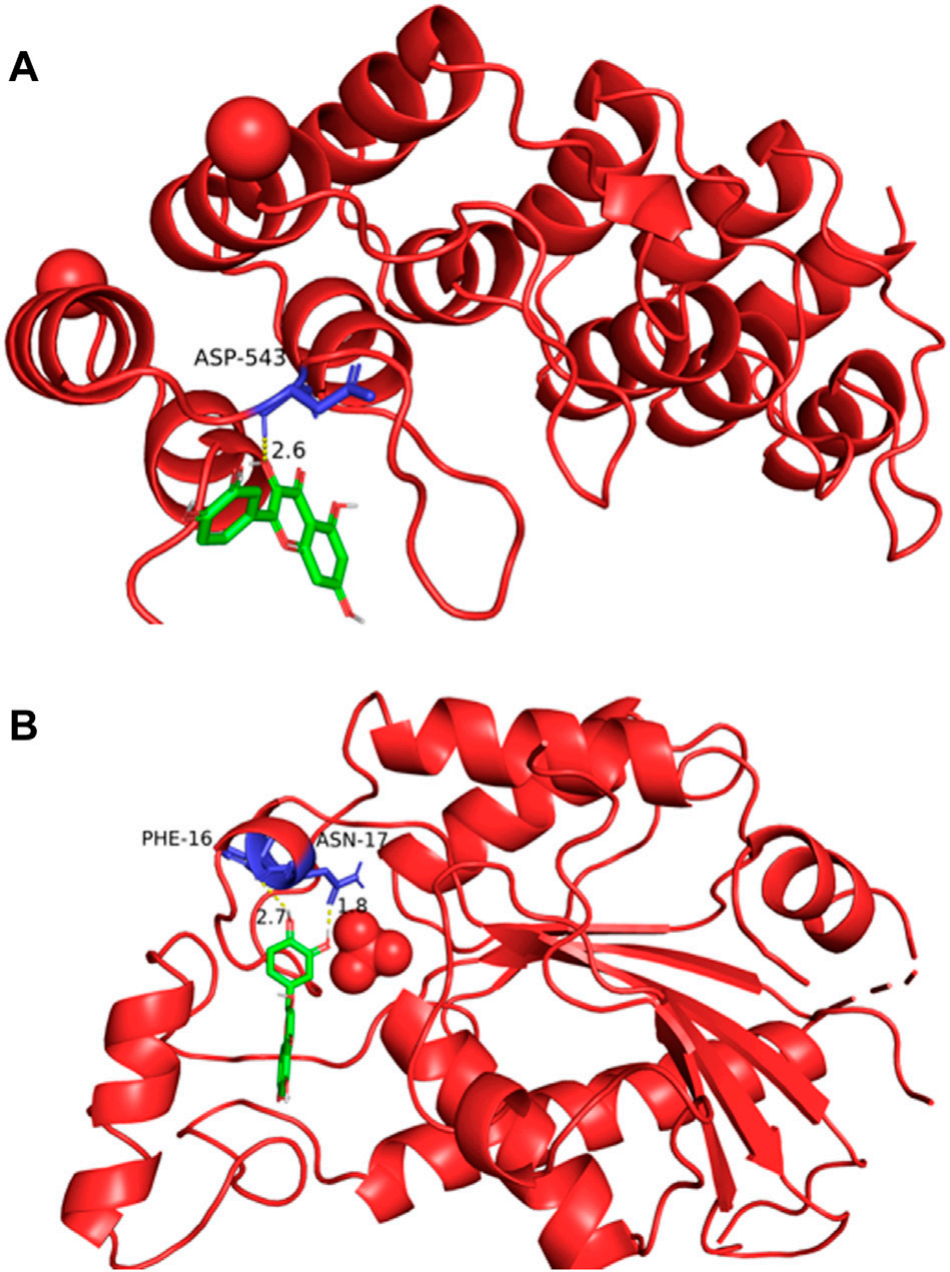
Macromolecular docking pattern diagram. **(A)** Docking diagram of quercetin and AKT1; **(B)** docking diagram of quercetin and TP53.

The potential targets of Guishao-Liujun decoction acting on GC were macromolecularly docked with the main compounds of Guishao-Liujun decoction calculated by the topological analysis using AutoDockTools-1.5.6, and the more stable the ligand-receptor binding confirmation was, the more likely the action occurred. Serine/threonine-protein kinase (AKT1) and tumor protein p3 (TP53) were chosen as the top two main targets based on the degree value. AutoDock Vina was used for macromolecular docking of the components, binding energies of -4.25 kcal/mol indicate some binding activity between the ligand small molecule and the receptor protein; binding energies of −5.0 kcal/mol indicate good binding activity between the two; binding energies of −7.0 kcal/mol indicate that the ligand and the receptor have strong conjugation activity (Hsin et al., 2013), and the binding energies of quercetin and the two core targets were −6.7 and −7.7 (kcal/mol), respectively. The specific docking results are shown in Figure 5.

## Discussion

TCM has exceptional anti-cancer properties, and in recent years, its anti-tumor mechanism has been of strong research interest. The etiology of GC in TCM is the deficiency in origin and excess in superficiality, that is, deficiency of the spleen is the origin, and heat, dampness, stagnation of qi, and blood stasis are in excess. Guishao-Liujun decoction is not only effective in nourishing the qi and blood, treating spleen, and stomach deficiency but also has a very significant function as an anti-tumor agent. In recent years, with the rise in the combination of TCM and Western medicine, many herbal medicines are playing a significant role in cancer treatment.

Through the collection and screening of the active components of Guishao-Liujun decoction, it was found that they mostly comprise eight compounds including kaempferol, quercetin, luteolin, etc. These components with their effective medicinal properties are the material basis of Guishao-Liujun decoction for GC treatment.

The major targets of Guishao-Liujun decoction for GC treatment are IL-17, AKT1, TP53, and TNF, as derived from the analysis of the protein interaction network (Kumar et al., 2021). Serine/threonine-protein kinase (AKT1) has a role in cancer cell proliferation, inhibition of apoptosis, tumor angiogenesis, and energy metabolism (Chen et al., 2020); IL-17 is closely linked to gastric mucosal atrophy, gastric precancerous lesions, and the degree of intestinal epithelial hyperplasia (Li et al., 2021); TP53, a common tumor suppressor gene, is involved in apoptosis or the regulation of cell cycle and is related to lymph node metastasis, tissue differentiation, and infiltration depth of GC (Wen et al., 2021); TNF is also one of the pro-inflammatory factors; and TNF-α can induce apoptosis through the nuclear factor kappa-B (NF-κB)-activated signaling pathway, caspase-mediated signaling pathway, and c-Jun N-terminal kinase (JNK) signaling pathway and it regulates the immune system to control the proliferation and apoptosis of tumor cells. By upregulating the serum TNF-α level and downregulating the TGF-α level, quercetin can increase the immune function of the body resulting in the suppression of tumors (Zhang, 2022), which is similar to the findings of the present study that the TNF signaling pathway is enriched. Nuclear factor κB (NF-κB) is a ubiquitous transcription factor of the NF-κB/Rel protein family with the ability to mediate immune stress and inflammatory response, etc. Recent research demonstrated that NF-κB is expressed highly in different tumor types, and its expression has a role in apoptosis inhibition, cell proliferation, invasive behavior, and angiogenesis (Sack, 2002; Ashrafizadeh et al., 2021; Huang et al., 2022). The molecular mechanism of anti-apoptosis in GC after NF-κB signaling activation involves the direct regulation of downstream target genes’ expression, such as through the regulation of B-cell lymphoma-2 (Bcl2) family proteins and caspase family proteins to exert anti-apoptotic effects. Moreover, NF-κB plays role in all phases of the inflammatory response by causing gene expression and stimulating cytokines’ release. The NF-κB pathway is a crucial connection between inflammation and GC (Gurevitch et al., 1997). It has been discovered that baicalin can exert its biological effects using different signaling pathways and has the potential to inhibit the inflammatory response by suppressing the TLR4/NF-κB signaling pathway. Toll-like receptors (TLR) are not only correlated with tumor growth and immunosuppression but are also involved in apoptosis and immune system activation, and the detection of molecules related to this signaling pathway may become a reference indicator for GC prognosis (Echizen et al., 2016; Susi et al., 2019; Zargari et al., 2022).

According to the aforementioned results, the active components in Guishao-Liujun decoction have effective binding activities with IL-17, TP53, AKT1, and TNF, but further validation is required for the bio-functionality of its compounds for the treatment of GC.

There are certain limitations to network pharmacology research: 1) the effective ingredient information sources in the traditional Chinese medicine database are limited, and cannot fully and timely reflect the substances found in traditional Chinese medicine. 2) Because the dosage of each medicine prescribed in Chinese medicine has a varied effect, the efficacy and concentration of Chinese medicine’s active substances are strong or weak. However, the most present research chooses beneficial compounds based on oral availability and drug-like qualities of chemical substances and rarely takes into account the dose-effect connection of Chinese medicine ingredients (Newman, 2020). 3) Traditional Chinese medicine comprises a complicated chemical composition system rather than a random assortment of substances. Its effect could be the overall effect of a set of effective components comprising numerous chemical components or the effect of its metabolites once they enter the body. As a result, it is unscientific to equate effective components, targets, and pathways with diseases, and it is vital to evaluate the holistic view of traditional Chinese medicine and the metabolic process *in vivo* from a clinical standpoint (Chen et al., 2022; Mei et al., 2022). 4) When existing network pharmacology analyzes the action targets of pharmaceutical ingredients, it can only predict using energy matching and compound geometric characteristics matching, but not the combined action type of the two, such as the target’s activation or inactivation state, the drug’s agonist or antagonist, and so on. 5) Traditional Chinese medicine (TCM) uses the concepts of “harmony between man and nature” and “therapy based on syndrome distinction” to treat ailments. The existing database of disease targets focuses primarily on the names of diseases in western medicine, rather than TCM syndromes, making it difficult to accurately reflect the internal relationship between TCM diseases and syndromes, and the theoretical foundation of TCM prescription construction (Wu et al., 2021). 6) The protein–protein interaction database is biased because it is based on a single source. Many academics, for example, frequently use STRING for online analysis, producing false positive or false-negative results. 7) Typically, research begins with a target protein that is shared by traditional Chinese medicine and diseases, and rarely considers drug ingredients in combination with other biological functional molecules, such as Chinese medicine ingredients-metabolites, Chinese medicine ingredients-lncRNA, Chinese medicine ingredients-circRNA, and so on. As a result, traditional Chinese medicine network pharmacology research has to be standardized and enlarged.

## Conclusion

In conclusion, this study explores the mechanism of action of Guishao-Liujun decoction in the treatment of GC by network pharmacology and assessed that Guishao-Liujun decoction works through a variety of targets and components in GC treatment. As an emerging field, network pharmacology is useful for drug development and clinical guidance, but there are many ambiguities in the process of target collection and investigation; therefore, a protein model is required to validate the experiments, and cellular and animal experiments are needed to improve the conclusions.

## Data Availability

The original contributions presented in the study are included in the article/Supplementary Material; further inquiries can be directed to the corresponding authors.
